# Trusted Threat Intelligence Sharing in Practice and Performance Benchmarking through the Hyperledger Fabric Platform

**DOI:** 10.3390/e24101379

**Published:** 2022-09-28

**Authors:** Hisham Ali, Jawad Ahmad, Zakwan Jaroucheh, Pavlos Papadopoulos, Nikolaos Pitropakis, Owen Lo, Will Abramson, William J. Buchanan

**Affiliations:** Blockpass ID Lab, Edinburgh Napier University, Edinburgh EH10 5DT, UK

**Keywords:** blockchain, smart contract, Hyperledger Fabric, privacy-preserving, Interplanetary File System (IPFS), threat intelligence sharing, MITRE ATT&CK framework, cyber hunting

## Abstract

Historically, threat information sharing has relied on manual modelling and centralised network systems, which can be inefficient, insecure, and prone to errors. Alternatively, private blockchains are now widely used to address these issues and improve overall organisational security. An organisation’s vulnerabilities to attacks might change over time. It is utterly important to find a balance among a current threat, the potential countermeasures, their consequences and costs, and the estimation of the overall risk that this provides to the organisation. For enhancing organisational security and automation, applying threat intelligence technology is critical for detecting, classifying, analysing, and sharing new cyberattack tactics. Trusted partner organisations can then share newly identified threats to improve their defensive capabilities against unknown attacks. On this basis, organisations can help reduce the risk of a cyberattack by providing access to past and current cybersecurity events through blockchain smart contracts and the Interplanetary File System (IPFS). The suggested combination of technologies can make organisational systems more reliable and secure, improving system automation and data quality. This paper outlines a privacy-preserving mechanism for threat information sharing in a trusted way. It proposes a reliable and secure architecture for data automation, quality, and traceability based on the Hyperledger Fabric private-permissioned distributed ledger technology and the MITRE ATT&CK threat intelligence framework. This methodology can also be applied to combat intellectual property theft and industrial espionage.

## 1. Introduction

It is mandatory for organisations to protect themselves from a wide range of cyberattacks that are constantly expanding. Legacy applications often introduce vulnerabilities and need to be considered now more than ever. As organisations struggle to keep their systems up to date, they also face major challenges in this digital era due to the increasing number and diversity of digital technological artefacts, as well as the broadening of the attack surface. The traditional methods for threat analysis are normally manual, which can be time-consuming and prone to errors [1]. Threat intelligence is a powerful tool that organisations can use to protect their digital infrastructures against new cyberattacks while generating meaningful reports. However, the effectiveness and efficiency of the existing threat intelligence solutions often vary in their ability to cover a wide range of attack tactics and techniques.

Slow advancements in threat intelligence sharing come from stakeholders who are resistant to providing their threat information due to the potential consequences of revealing their private data. Attracting and convincing stakeholders to employ threat intelligence technologies is, thus, a current priority. Thus, the trust and quality of threat information sharing are often important factors in convincing users and stakeholders to share their private data. In addition, other factors are relevant to the scale-up of the data quality that can attract stakeholders and practitioners to apply threat information sharing in their organisations. These metrics may include dependability, correctness, and timeliness. Therefore, by having such system qualifications, practitioners can generate high-quality automated and meaningful reports about existing and emerging threats that can help reduce the risk of possible threats. Organisations cannot defend themselves in isolation from the threat landscape due to the emerging threats [1]. However, threat information sharing raises a massive concern about creating a trusted system around validity, security, privacy, and traceability.

Conventional network systems for information sharing are often centralised and controlled by a single entity over the Internet using a location-based approach (URL—Universal Resource Location) to reach resources. As a result, data are often vulnerable to a single point of failure and are prone to a denial-of-service attack (DoS), which represents the major concern of the centralised networking system [2]. In contrast, a decentralised system, which is the core concept of our proposed infrastructure (an infrastructure is the set of components), is leveraged to prevent centralised system problems. For example, DLT (distributed ledger technology) is a decentralised system that can increase data security without requiring the intervention of a third party. Data are then handled in a distributed manner. Furthermore, any participant nodes can have a replica of a global ledger.

With a public ledger, no permissions are required for new users to join the network. To restrict access, we can use a private blockchain, where only authorised users can read, write, and be engaged in the consensus process to validate the new transactions. Table 1 shows a comparison of different DLT types.

Using Hyperledger Fabric, threat intelligence can be shared and stored in a sharding manner through a secure replication to all nodes. This is delivered over a reliable, scalable, and efficient infrastructure. Additionally, the private and permissioned nature of Hyperledger Fabric supports pluggable and flexible identity systems, distributed smart contracts, and a permissioned-access control approach to private data [3]. The permissioned approach ensures that private information stored in the ledger is not accessible to unauthorised users and that only approved participants can access it using their authorised identity certificates [4]. On this basis, one can enhance privacy-preserving methods for threat information sharing.

### 1.1. Problem Statement

This section outlines some of the concerns around areas of threat analysis and sharing.

#### 1.1.1. Centralisation of the Public Key Infrastructure

The issues associated with the centralisation of the public key often develop when the participating node volume increases and users attempt to execute huge transactions simultaneously, which may cause bottlenecks and reduce the system’s scalability. To address this issue, we suggest the use of decentralised identities [5].

#### 1.1.2. Trust and Lack of Interest

Establishing a threat intelligence sharing partnership demands stakeholders’ trust [6,7]. Trust is critical to a cyber threat intelligence (CTI) exchange ecosystem and is challenging to maintain. A third party is often used to establish a trusting relationship between stakeholders. In contrast, this method can be prone to leaking sensitive information. Threat intelligence sharing may include information that should only be disclosed to trusted partners or to no one, such as information related to personally identifiable information (PII) unrelated to creating situational awareness [8]. The lack of interest and sharing is seen to impact organisational security negatively, which represents a major concern. Stakeholders, too, often do not trust threat information sharing infrastructure, which might reveal their sensitive data. Consequently, a new threat information sharing framework could provide privacy-preserving mechanisms based on private and permissioned DLTs through a smart contract without a trusted third party involved to address these concerns.

#### 1.1.3. Privacy and Anonymity

Despite the fact that a threat-sharing infrastructure is commonly provided by a third party, it is often deemed insecure where anonymity is critical for cooperation [7]. Organisations must thus prioritise user privacy by counter threat intelligence with trusted stakeholders or anonymising data to maintain data privacy. Thus, data anonymity using threat intelligence and encryption techniques is suggested to ensure secure data-sharing and that data are unrevealed to unauthorised users.

#### 1.1.4. Legal Consideration

Another critical part of threat information sharing is understanding any legal issue [9]. Therefore, before disclosing any information, one must be aware of all applicable rules and regulations to prevent the committing of an offence or unintentionally engaging in illegal activity. For example, threat intelligence sharing can contain personally identifiable information, and sensitive data should be often sanitised before exchange. In addition, executive and legislative bodies can restrict the information that an organisation shares. Appropriate restrictions are necessary, but unreasonable ones often reduce the quality of shared data.

#### 1.1.5. Data Quality

Data dependability, correctness, and timeliness are all factors that contribute to high quality. Because cyber security cannot rely on outdated and inaccurate data, maintaining data quality is critical. When the number of stakeholders and associated data sources often grows over time, quality issues develop [10,11]. Instead, before acting on threat information, an organisation must ensure that it is authentic and relevant, along with the risks being properly identified [6].

#### 1.1.6. Lack of Automation

The lack of an automated sharing mechanism and data quality both act as obstacles to information sharing [11]. Unlike modern technologies, the most traditional approaches to the analyses of threats are done manually, which raises considerable concerns over inaccurately and erroneously generated results. Thus, one can address this issue by using automated threat technologies to get adequate threat assessment results. Therefore, we proposed using automated threat analysis instead of the conventional approaches.

#### 1.1.7. Inevitable Costs

Using computationally expensive resources makes monetary and personal information sharing quite unattractive. Commercial threat analysis solutions are often costly and difficult to manage, while open-source alternatives can be hard to manage. According to Ponemon’s investigation, overall running costs are cited by 21% of respondents as a factor for not engaging in an exchange program [12]. As a result, organisations that prefer not to engage in exchange programs can slow down the efforts to share information, which is often fundamental to cyber security. Our approach aims to address these shortcomings and encourage information sharing.

### 1.2. Contributions and Paper Structure

The following are the primary contributions of this paper:

- Creating a new framework based on permissioned blockchain technology for privacy-preserving and trustworthy threat intelligence sharing.
- Demonstrating how threat intelligence is conducted in practice: analysing the detected threats and generating meaningful reports while increasing data quality.
- A proof-of-concept implementation of the proposed solution along with a security analysis to verify the feasibility of our solution.
- Performing experiments with Hyperledger Fabric and measuring the network capabilities during transaction execution: transaction rate, latency, and throughput.

The rest of this paper is organised as follows: Section 2 briefly presents the background needed around threat intelligence, blockchain technology, scalability, the Interplanetary File System (IPFS), and Hyperledger Fabric. Section 3 provides references to the related literature while discussing the existing data sharing solutions. Section 4 presents the proposed solution while covering a wide range of problem statements and system executions. Section 5 details our implementation by presenting data logs, threat analyses, threat file encryption and storage, and threat information sharing technologies. The security consideration, anonymity consideration, and latency scalability throughput are discussed in Section 6. Finally, Section 7 draws the conclusions while providing some pointers for future work.

## 2. Background

This section introduces the essential technologies and principles of our proposed solution while focusing on the most effective tools related to threat analysis. We provide a quick overview of threat intelligence, blockchain and Hyperledger Fabric, the creation of an efficient way for threat sharing, a privacy-preserving method, and a proactive defensive threat analysis tool, and Big Data processing.

### 2.1. Cyber Threat Intelligence

In the threat landscape, threat hunting, cyber threat intelligence (CTI), and incident response (IR) are deeply correlated, although they often use different techniques and procedures to achieve their goals. Threat intelligence is also known as cyber threat intelligence (CTI). CTI can be thought of as a starting point for threat hunters. The next step after security breaches have been detected is incident response.

Threat intelligence is evidence-based information about existing or emerging threats or hazards to assets, including context, mechanisms, indications, consequences, and actionable recommendations, which may be used to make decisions about an organisation’s or individual’s reaction to that threat or hazard [13]. Sharing threat intelligence is seen as a proactive way to defend against attacks and improve an organisation’s overall security by making it easier to predict attacks and stop them before they happen. Threat intelligence technology is used to analyse adversarial behaviour based on evidence, since it must be trusted. The threat intelligence life cycle often includes a sequential process that starts by detecting threat data and classifying, analysing, and sharing them. As a result, it can generate informative reports about historical and new threats aimed at an organisation’s assets [14], which may help them mitigate the risk of a cyberattack. Threat intelligence can support the automated and comprehensive vision based on accumulative knowledge. Consequently, it gives detailed insights into factors such as the nature, motive, timing, and how an attack could be carried out. As a result, threat intelligence helps organisations and companies make quicker, more informed security options and upgrade their behaviour from reactive to proactive in order to combat the threat [15].

### 2.2. Blockchain

Blockchain is a decentralised, distributed ledger technology that has gained a solid reputation among current security techniques, especially in increasing trust levels for transactions. As a result, it has been implemented with great success in systems that respect privacy [16]. Blockchain was initially built for immutable (tamper-proof) records to protect bitcoin transactions from alteration and to ensure transaction integrity. Additionally, it has gradually proven both transaction validity and system efficacy in various areas. There are key features that make blockchain a good candidate for various security applications, such as features of immutability, transparency, and traceability, while providing a trusted method whereby verification and validation are done without the need for trusted signers or third parties. To achieve the latter, blockchain requires consensus algorithms [17]. Although most of blockchain’s characteristics are beneficial, it still has some shortcomings in scalability, latency, and privacy concerns. Blockchain, too, can have limited data storage and a small processing capacity. Moreover, the consensus mechanism is relatively slow due to the time-consuming negotiation process amongst nodes to add a new transaction block to the chain. All blockchains, permissioned or permissionless, have specific operational phases, which follow the *order–execute* pattern. Regarding the transaction execution process, the blockchain follows the *order–execute* pattern. With this, the transactions are executed in sequential order, which is considered the main reason for bottleneck issues that occur when the number of transactions increases. Additionally, these issues contribute to decreased throughput and increased delay. The use of an external off-chain data store can help to tackle some of the blockchain scaling concerns. With this, blockchain systems could be integrated with other technologies to enable large data scales, such as double blockchains or by using the Interplanetary File System (IPFS) to store data off-chain.

### 2.3. Scalability Solution

Most blockchains are incapable of handling large amounts of data because of their limited storage space on the chain. The most common solutions involve the following.

#### 2.3.1. On-Chain Storage

We might think that it is normal to execute data on the blockchain. However, blockchain often has limited storage capacity, and this is referred to as on-chain data. As a result, it may be necessary to seek an external solution in order to store large amounts of data. However, it is impossible to analyse large amounts of data and maintain them on the chain without increasing storage capacity. Some alternatives for dealing with big data, such as storing information off-chain using low-cost open-source approaches, are compatible with blockchain technology. This includes using a double blockchain and the IPFS.

#### 2.3.2. The Interplanetary File System (IPFS)

The IPFS is an open-source, peer-to-peer file protocol that has a decentralised nature. It is frequently used in combination with blockchains to store data off-chain. When used with a blockchain, the IPFS shows a significant capability of storing large amounts of data in an off-chain manner. Within the IPFS, data are stored in a distributed manner using key-value data storage management [18]. In the IPFS network, any participant node can retrieve the data due to the IPFS’s public-nature network. One can maintain data confidentiality by using encryption techniques to protect data disclosure by an unauthorised user. In other words, users can access stored threat information, retrieve private data, and then decrypt them if the user is authorised to do so. In addition, the IPFS has the key advantage of storing files in a distributed manner, which provides a more resilient approach to storing data than conventional centralised approaches.

#### 2.3.3. The Content Identifier (CID)

The initial step of the IPFS’s mechanism starts when a file is uploaded and then split into blocks or chunks. Each chunk is associated with a unique identifier called a *Content Identifier (CID)*. The CID represents a unique value or hash-key of content inside the IPFS. A CID is a 256-bit self-describing [19] identifier. The following is an example of a CID.

*QmQwhiK1YqjSswTJTLcYiA6TtnQrbua3aceeqEbeK8s2sP*

This value is highly likely to differ whenever the content changes. In addition, the IPFS uses the CID to share and retrieve the file content from different places that represent the content’s addresses.

#### 2.3.4. Off-Chain Storage

Off-chain refers to the capacity to transport, store, and process data outside the blockchain. Smart contracts running on a blockchain can engage with off-chain data and carry out sharing activities. Using the IPFS, one may deal with two different approaches. The first is when data are transferred off-chain and used in the IPFS, and then moved back on-chain and committed to the blockchain via a consensus mechanism. Furthermore, off-chain transactions are recorded on a side-chain through distributed ledger technologies, thus enabling quick execution. For example, such an approach is reasonable when we need to execute 10,000 fast transactions, combine them into one transaction, and then bring that one transaction back on-chain to be submitted to consensus and committed to the blockchain. Notably, a Merkle tree combines the transactions into a single transaction, which is then posted on-chain for a consensus and commitment to the primary blockchain [20]. Merkle trees are a data structure that maps objects and links the contents to their address. Thus, it can use a Git repository as a version control system and to exchange objects. Another acceptable approach is when transaction data are stored on-chain and the original data are saved off-chain. One can then obtain the content’s CID, which specifies a content address within the IPFS. Then, the CID is sent to the targeted users. As a result, the trustworthy user retrieves the data from the IPFS using the obtained CID.

### 2.4. Components and Workflow of Hyperledger Fabric

Hyperledger Fabric is a private-permissioned blockchain framework that was chosen to develop a *proof of concept* for threat information sharing that aims to share threat information efficiencies amongst trusted organisations. An organisation needs to share threat intelligence by communicating the data securely over encrypted or private channels or a trusted infrastructure to upgrade their threat detection capability. However, these channels must be tamper-proof and allow access only to trusted partners. Hyperledger Fabric has been developed to be extremely modular, scalable, and general purpose, offering privacy, confidentiality, and scalability. Hyperledger Fabric’s operation phases include *execute–order–validate* patterns rather than *order–execute*, as in the blockchain. The blockchain has several concerns related to scalability, latency, and throughput. On this basis, this novel pattern of *execute–order–validate* is used to address the limitations of the *order–execute* approach mentioned above. Additionally, the privacy policy in Hyperledger Fabric is basically applied in two main components.

#### 2.4.1. Chaincode

Chaincode represents the Hyperledger Fabric’s smart contract, which is executed in a distributed manner on nodes if business logic is met. In other words, chaincode includes the business rules that govern trusted partners’ interactions. Java, Go, and JavaScript are all general-purpose programming languages that can be used to write chaincode.

#### 2.4.2. Endorsement Policies

Endorsement policies are rules that describe which peers can perform transaction endorsement and approve a particular chaincode invocation. Endorsing peers manage the validity of chaincode execution findings by giving a signature for those results. These policies govern all public and private data in the world state database. A collection-level endorsement policy may control private data collection specifically. The endorsement policies are expressed logically as *AND (Org1, OR Org2, OR Org3)*. Examples of endorsement policies include the following: *1-of-any* means that any node from any organisation can endorse the proposed transaction, *and 2-of-any* means any two nodes of an organisation can endorse the proposed transaction through their validation of their identities. Hyperledger Fabric’s private and permissioned nature provides a flexible identity system, chaincode, and a robust privacy policy for accessing private data. It assures that only authorised users have access to private data by requiring them to provide their identity certificates, which are issued by the membership service provider. Hyperledger is a private and permissioned modular architecture distinguished by its pluggable components, including a membership service provider, chaincode, and consensus.

### 2.5. Transaction Workflow through Hyperledger Fabric’s Architecture

**Figure 1:**
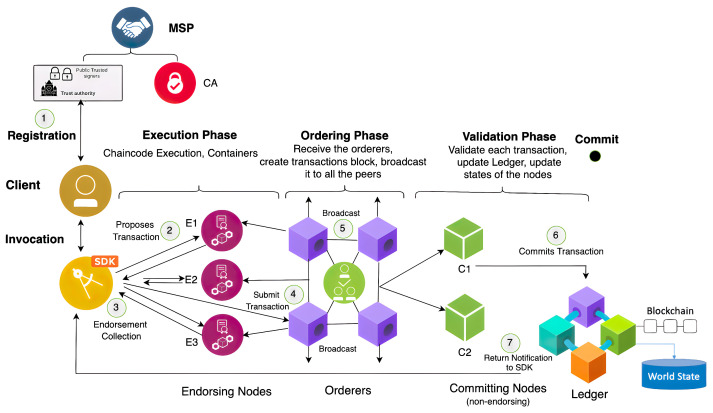
Hyperledger Fabric’s transaction flow.

Hyperledger Fabric’s architecture (architecture is a word used to describe the way in which a collection of parts function as a whole) consists of multiple organisations, each of which includes a number of peer nodes, which run smart contracts known as chaincode. Furthermore, the chaincode can query ledger data, endorse transactions through the endorser’s nodes, and interact with applications. To execute a transaction, a unique identifier known as a “txid” is issued, and it should have a creator known as a “transaction creator” [21]. The Hyperledger Fabric has three phases: *execute*, *order*, and *validate* patterns, as illustrated in the transaction workflow shown in Figure 1. (1) The membership service provider (MSP) validates all identification certificates for network users. (2) The authenticated user first sends the transaction proposal to be endorsed. (3) The endorsed transactions will be collected (4) and then submitted to an ordering service component through the channel. (5) The ordering service includes the consensus mechanism that is held on the orderer nodes. Orderer nodes do not have smart contracts or ledgers; instead, their nodes are in charge of validating the transactions, ensuring consistency, and ordering the transaction sequence inside the block. Validation can be performed when the orderer service broadcasts the validated transactions to all network peers and membership service providers who issued their identities to be a certificate authority. All peers validate the received transactions and can then add them to the new block. (6) Each peer will use the execution results to update the world state if all transactions are acknowledged. Each peer adds the new block to its local blockchain when all transactions have been validated, (7) and then the notifications will be sent to the SDK (software development kit).

### 2.6. Hyperledger Fabric’s Features

Hyperledger Fabric includes several features. The following are the most notable among them:

1. *Permissioned blockchain:* Unlike a public blockchain, where nobody needs permission to join the network, Hyperledger Fabric requires an identity and certificate authority for any user or node to join the network [22].
2. *Privacy and confidentiality of transactions:* Channels allow a subset of nodes through the anchor node to link different organisations, which make up the consortium. The ledger of a channel can be accessed only by organisations that are part of the channel. Therefore, participants can only see network features based on this aspect.
3. *Highly modular and configurable architecture:* Hyperledger Fabric enables plug-and-play ordering, membership, endorsement, and validation services. A pluggable consensus algorithm also improves the platform. The ledger supports the LevelDB and CouchDB databases [22].
4. *Efficient data query:* By using CouchDB, it could execute queries that are more efficiently compared to other relational databases with less latency and with more simple queries.
5. *High-transaction-throughput performance:* Hyperledger Fabric is scalable. Peer nodes are liberated from ordering (consensus) responsibilities, while transaction execution is independent of ordering and commitment. The division of labour relieves the ordering nodes of transaction execution and ledger maintenance.
6. *Low latency of transaction confirmation:* Hyperledger Fabric is considered the fastest amongst all the permissioned blockchains and can be generated within only a few organisations, thus contributing to a reduction in the latency. Furthermore, it does not have a mining process as in a blockchain, which it makes the system fast when verifying and committing the transactions.
7. *Offering multiple languages in which to write smart contracts:* One can write smart contracts in different programming languages, such as Java, Go, or Node.js [22,23].
8. *No cryptocurrency:* Unlike public blockchains and several other technologies, Hyperledger Fabric does not involve a cryptocurrency [22].

## 3. Related Work

In the work of Bawane et al. [24], the authors introduced the Ethegram architecture to address common social media issues caused by system centralisation. The authors employed the Ethereum blockchain and the IPFS (Interplanetary File System) peer-to-peer file system to disseminate the stored material and overcome single-point-of-failure problems. Furthermore, the proposed decentralised network addresses centralised system concerns by utilising scattered peers rather than relying on a trusted intermediate. However, because the selected blockchain contains a cryptocurrency, adoption of this architecture may be difficult. As a result, transactions may be costly.

In the work of Havelange et al. [25], the authors established the LUCE framework, a novel means of data management that considers data accountability and licensing conditions. This system makes use of a blockchain ledger to incentivise data sharing and reuse by making it easier to comply with licensing restrictions, such as registering data consumption and purposes. The primary goal of developing the LUCE framework was to enable individual data to be amended and destroyed following legislation such as the GDPR’s rights to access, alteration, and deletion. This effort is analogous to our proposed infrastructure regarding GDPR compliance, licensing conditions, and rewarding data sharing; however, it does not consider using the IPFS to scale up data storage to address scalability issues.

In the work of Wang et al. [26], the authors developed a unique architecture to address difficulties with data sharing, such as security, openness, and traceability. The proposed approach allows for the simultaneous usage of two blockchains. One blockchain is used to store the original data, while the other is used to store the transactions. This architecture aims to securely transfer data while also providing characteristics such as linkability and traceability. Furthermore, this infrastructure is connected with another proxy encryption technology to improve the system’s privacy and security. This article advocated for the use of dual blockchains to address the previously identified difficulties; nevertheless, this alone is insufficient to protect the confidentiality of the stored data, especially as this system was designed to enhance the traceability of the stored data.

In the work of Politou et al. [27], the authors merged the blockchain and IPFS technologies. They integrated a blockchain with the IPFS data sharing technology to provide a larger data scale and off-chain storage of personal data. To prevent data censoring and comply with the General Data Protection Regulations (GDPRs) and the Right to be Forgotten (RtbF), the original data are shared and replicated through IPFS nodes rather than the blockchain. The European Union adopted the GDPR to safeguard personal data within the EU and any linked businesses in the same context.

In the work of Preuveneers et al. [28], the proposed system was called TATIS. With TATIS, only authorised users can access sensitive data throughout their transport to and from other threat intelligence systems. This paper has been fully implemented in a distributed manner atop the Malware Information Sharing Platform using distributed ledger technology (DLT) to control access to CTI data, share encrypted CTI data (based on the CP-ABE cryptographic scheme), and account for and manage CTI data provenance in order to reinforce trust (MISP).

In the work of Grundstrom et al. [29], their proposed system included an anonymous protocol that adheres to the IPFS concept of removing any personally identifiable information (PII) from data distributed amongst IPFS nodes and ensuring that an erasure request reaches all nodes via the delegated deletion method. Aside from that, only the owner or their delegates can delete the original material. As stated by the authors, this method for erasing data does not affect system efficiency; therefore, it offers strong security and adequate performance for producing content-dependent keys.

We conclude that most previous and existing works employ a centralised network system or public-blockchain-based infrastructure to share information and increase confidentiality. However, unlike ours, these works do not support the large data scale and privacy-preserving threat information sharing addressed in our work.

Table 2 shows how our work stands apart from the rest of the existing results in the same context. It proposes combining private-permissioned blockchain technology with the IPFS to store and share threat information between distributed peers. This combination assures the anonymity of the stored content while also providing outstanding performance in network latency, transaction throughput, and central processor unit (CPU) and memory use. Along with that, we deploy a decentralised identity management system to assure distribution identification in a safe, trustworthy, and accessible manner.

The MITRE ATT&CK framework is an open-source threat intelligence tool that involves a comprehensive knowledge base of cyberattack tactics and techniques gathered from actual observations of adversary behaviour that has already affected organisations in the past [30]. The MITRE ATT&CK framework is compatible with the other infrastructure components that we propose. It uses the STIX language for information sharing to enable organisations to share threat intelligence with their trusted partners in a manner that enhances their defensive capabilities, including collaborative threat analysis, automated threat information sharing, automated detection and response, and more [31]. The MITRE ATT&CK threat information format is a JSON file that consists of tactics, techniques, and mitigation in the form of IDs. It provides a variety of colours and scores that can be used to implicitly, concisely, clearly, and comparably depict the attackers’ behaviour. The threat match-up depiction is retrieved in JSON file format and shared with a trusted end-user. The MITRE ATT&CK navigator serves as the user interface for trusted partners, allowing threat information sharing to be examined and contrasted to identify mitigation and effective defence points. A partner is defined by their node identification in order to download the sharing file on the local system and then easily upload, evaluate, and compare the threat dataset on the MITRE ATT&CK navigator.

## 4. Proposed Solution

This section demonstrates the techniques used in our research, along with the configurations and decision making. We also refer to the implementation details that drove our tests on the network model.

### 4.1. Outline

We used the MITRE ATT&CK framework, Hyperledger Fabric, and the Interplanetary File System for threat analysis and sharing across a trusted infrastructure. This infrastructure enables organisations to communicate information with one another quickly and makes the process more flexible, automated, and secure. Due to the necessity of implementing threat intelligence sharing in our organisations, the criteria listed below reflect solutions that, ideally, solve challenges and attract stakeholders.

#### 4.1.1. Security and Trust

Private data may leak while traversing the public network when sharing threat information. Consequently, utilising a public blockchain system to share sensitive information is improper, insecure, and untrustworthy, since any participant node may reveal private information. Thus, the private and permissioned nature of Hyperledger Fabric provides flexible identities, enforces privacy policies, and implements smart contracts to ensure that unauthorised users do not access sensitive data, which are only allowed for those identified as authorised users [22].

#### 4.1.2. Business Logic

Hyperledger Fabric’s smart contracts, as previously stated, are known as chaincode and are used to handle business logic [23]. As a result, organisations may perform transactions by executing their business logic in any general-purpose programming language.

#### 4.1.3. Integrity and Accountability

Data need to be protected from unauthorised access, modification, or deletion. Maintaining data integrity ensures privacy and prevents unauthorised users from altering the data. In threat information sharing, unauthorised parties should not be allowed to modify, delete, disclose, or damage threat information. On this basis, using immutable hashed blocks in a blockchain and decentralised storage can ensure data integrity [22].

#### 4.1.4. Privacy and Confidentiality

Private channels are one approach to achieving privacy and confidentiality in Hyperledger Fabric [22]. They are communication routes that are exclusive to specific subgroups of network members and may only be used for transactions between those subsets of network users. Sharing threat information requires data privacy. To comply with the need-to-know principle, organisations must share threat information with partners. Our approach allows multiple parties to communicate through channel-based division, sharing different kinds of threat data while utilising the same infrastructure.

#### 4.1.5. Governance

Governance involves managing technical artefacts and controlling data and smart contracts inside a blockchain network [32]. Governance models are a vital technical competence. Policies in Hyperledger Fabric define these models. A policy specifies who may invoke a chaincode or add an MSP (membership service provider) to a channel. They are adaptable and may be configured as required.

#### 4.1.6. Availability

Organisations may need to quickly share the new threat information with trusted partners, which could help them respond to any potential similar cyberattacks in a timely manner. On this basis, it is very important that data and the network are available at the right time. Hyperledger Fabric is appropriate for ensuring ledger availability due to the distributed ledger and decentralised nature [22].

#### 4.1.7. Efficient Processing

Hyperledger Fabric provides synchronisation and parallelism by dividing network responsibilities based on the node type [22]. This feature alone will improve network speed by threading transactions for quicker processing in a Fabric network. As a result, only a subset of the network has to know the business logic, which frees up resources for the rest of the network to use. One does have to make it available to everyone on one’s network if one does not need it.

#### 4.1.8. Anonymity Consideration

Hyperledger Fabric contains an MSP entity for identity management, which can provide transaction anonymity by default. Decentralised identities are regarded as more secure than centralised identities. Additionally, the client’s private key is encrypted and signed by a trusted third party. This makes the data transactions private and keeps them from being revealed (identity management). It is also important to note that, as part of our design, threats (JSON) are encrypted before they are stored in the IPFS. This makes the data more secure and anonymous. TLS also establishes a secure channel for encrypted communication between parties. An authorised user should collect enough endorsements to submit the transaction for validation [22], which is regulated by the endorsers’ rules, without releasing or revealing the transaction details.

#### 4.1.9. Modular and Extensible

As previously stated, Hyperledger Fabric has a modular and extensible design that allows it to adapt to changes while sharing common components with other networks [22]. With a modular design, Fabric will become a blockchain architecture that any industry in the public or private sector may use.

#### 4.1.10. Latency

In the blockchain, a consensus mechanism is used to add blocks to the chain. Consensus is the mechanism used to verify the new transactions to be added to the chain, which takes time for agreement and causes transaction delays. To shorten this period, the consensus negotiation time must be reduced. One can find a takeaway for this issue, since Hyperledger Fabric has three execution phases; each is separate from another, rather than using two order–execute patterns, as in a blockchain [22].

#### 4.1.11. Caliper Benchmark Tools

Caliper is an open source software tools that is used to measure the Hyperledger network performance during transaction execution through several indicators such as send rate, latency, throughput and more [33]. We can specify the number of parameters against which the platform will be tested.

#### 4.1.12. Automation

Cyberattacks are often time-sensitive and require excessive automation [28]. Therefore, data automation must be done quickly, depending on standardised and structured data. The key aim of exchanging data in a structured way is that machines can easily use them. The threat intelligence file standard that we used in our solution was in JSON format. We used this standard when we transferred threat behaviour to tactics, techniques, and procedures (TTPs) by using the MITRE ATT &CK framework, then encrypted it and stored threat information in the distributed ledgers of the IPFS. Another feature of our solution is chaincode, which makes it easier to do important things for one’s business, as we used node.js.

### 4.2. System Design

**Figure 2:**
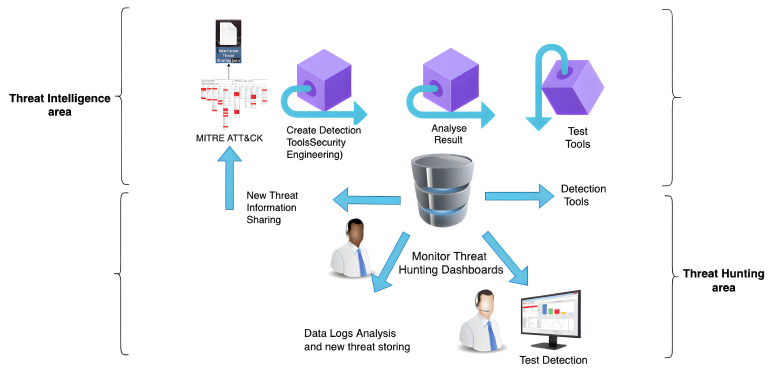
Threat analysis.

**Figure 3:**
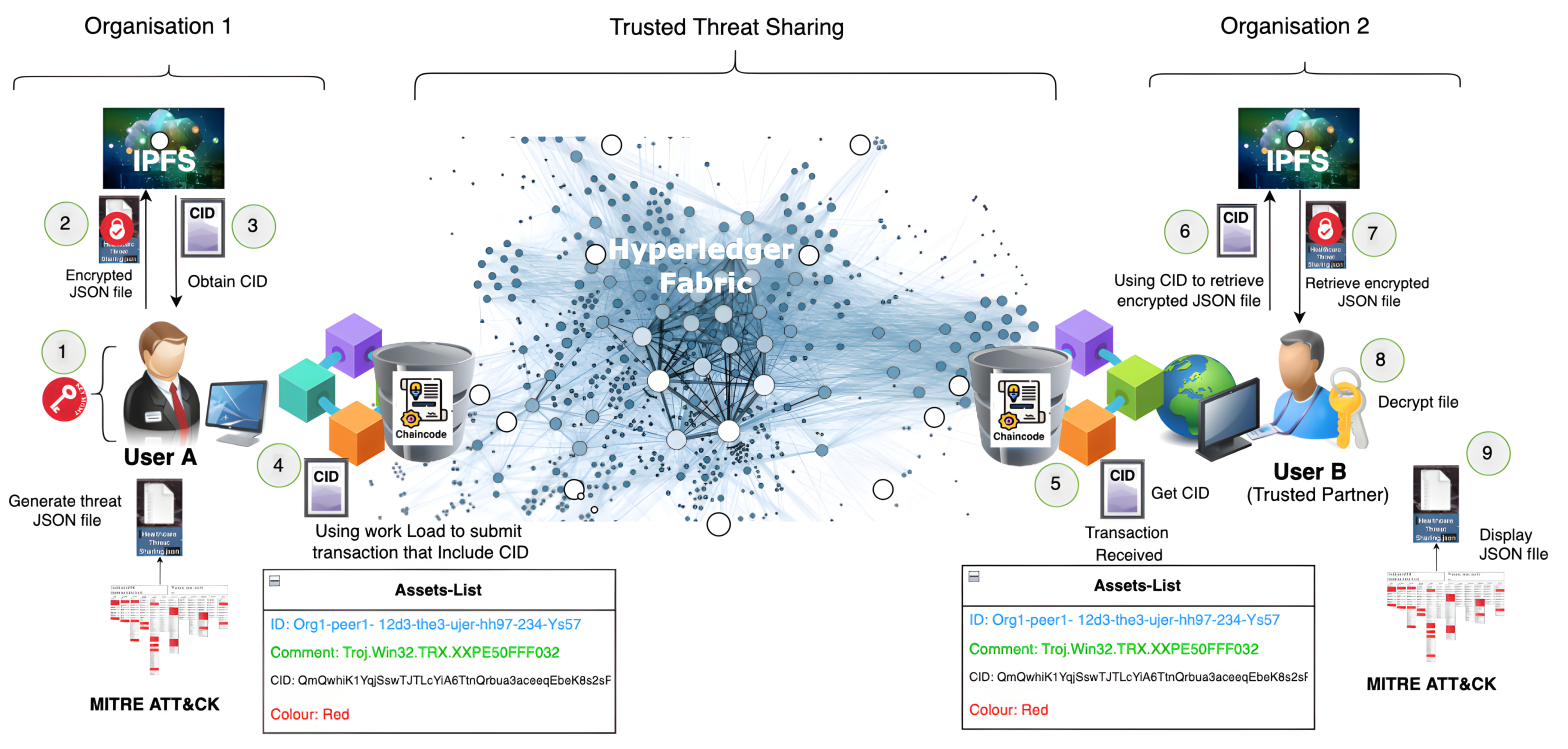
New threat information sharing through Hyperledger Fabric.

Organisations use threat intelligence approaches to develop their threat-hunting methodologies, with MITRE ATT&CK serving as a good tool and foundation of their systems. Threat intelligence methodologies differ from threat-hunting methodologies, but they can make them more effective and help them achieve their goals more quickly. The fundamental goal of threat hunters is to reduce the dwell time, which is the time frame between when the attack happens and when it is detected. Threat information sharing involves data processing, which starts with bringing the detected behaviours from data logs, and then classifying and mapping them to MITRE ATT&CK tactics and techniques, as shown in Figure 2. The MITRE ATT&CK framework contains cumulative comprehensive knowledge based on previous cyberattack tactics and techniques derived from attackers’ behaviours [34]. MITRE ATT&CK is a well-known threat intelligence tool that is used to analyse threat events and create helpful information reports. Furthermore, the MITRE ATT&CK framework can provide threat intelligence data in multiple file formats, such as JSON, SVG, and Excel [34]. As a result, upgrading the system detectability for emerging threats and sharing them with a trusted partner might be valuable, particularly for the organisation itself, as shown in Figure 3. As a result, JSON is used due to its nature as an open standard, ease of interpretation, and popularity in web technology. Then, the file is encrypted using self-encryption in the following step. Before storing and saving the threat information file in the IPFS, it is divided into chunks and encrypted using AES 256 or any other sort of encryption technique.

To utilise the Hyperledger Fabric network, a peer must be a member. Furthermore, the user’s node must be a registered member and have an identity through the MSP to be able to use the Fabric network. Figure 3 presents the detailed workflow of the implemented framework between the trusted users, starting with the threat intelligence tools for preparing meaningful information about a new or existing threat, file encryption, off-chain storage in the IPFS, and, finally, sending the threat information by traversing Hyperledger Fabric’s network infrastructure.

#### 4.2.1. Threat File Encryption

We proposed the use of encryption techniques that apply a sort of data anonymity, namely, self-encryption. Self-encryption is a type of convergent encryption that includes an obfuscation stage. It is a one-of-a-kind encryption method, since it has no independent keys, unlike other encryption methods, which typically have a separate public key and private key. It encrypts by using its file as the key. In this study, we proposed the use of the Maidsafe [35] self-encryption library.

The threat file (JSON file) generated by MITRE ATT&CK is encrypted via self-encryption, in which the file is divided into many pieces that are encrypted and stored in the IPFS, as explained in the following steps: First, the file is divided into at least three chunks, with the number of pieces increasing as the file size grows. Then, using AES 256, each file chunk is encrypted. Finally, the file chunks are obfuscated by performing an XOR operation between an encrypted chunk and another chunk’s randomly selected hash value, omitting its hash value.

#### 4.2.2. Off-Chain Threat Storage

It would be quite costly to keep the threat information sharing events that we would like to share between the trusted partners on-chain. It is vital to store threat information in a decentralised and controlled way. Hyperledger Fabric comes with CouchDB, a key-value database that allows the storage of a large amount of data, but it is still not big enough for big data, unlike the IPFS.

The IPFS can indeed be integrated with a distributed ledger technology, and it can also store data in an encrypted format off-chain. Despite all of the features that the IPFS has, it has privacy concerns. Because the IPFS is open to the public, anyone who joins the network can access any content saved in the IPFS and retrieve it.

To address the aforementioned problem, we used data anonymity for storage off-chain in the Interplanetary File System (IPFS). Data are encrypted before uploading to the IPFS to avoid unauthorised disclosure and prevent information from being transferred to unauthorised users. The sender can use a smart contract through the instantiate and invoke functions to share the CID, representing the encrypted content address inside the IPFS. The intended receiver can invoke the chaincode by using their authorised identity certificate to get the CID.

#### 4.2.3. Threat File Storage and Retrieval Steps

There are two users engaged in this implementation: A and B. A is a sender who uploads the file, and B is a receiver who requires the file. They must both be able to use the IPFS to download or retrieve the file. In our case study, we used a self-encryption library. Notably, self-encryption is just a choice, but one could use another encryption technique. The main purpose of encryption is to encrypt the threat information files (JSON) before they are uploaded to the IPFS to maintain privacy due to the IPFS’s public nature. In self-encryption, both the file chunks and the data map should be uploaded to the system.

**Figure 4:**
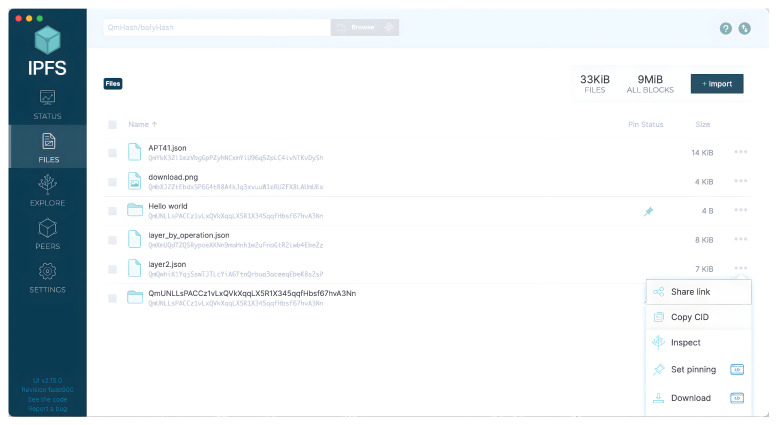
Files containing threat intelligence are shown here, along with the IPFS locations from which their respective CIDs were generated.

**Figure 5:**
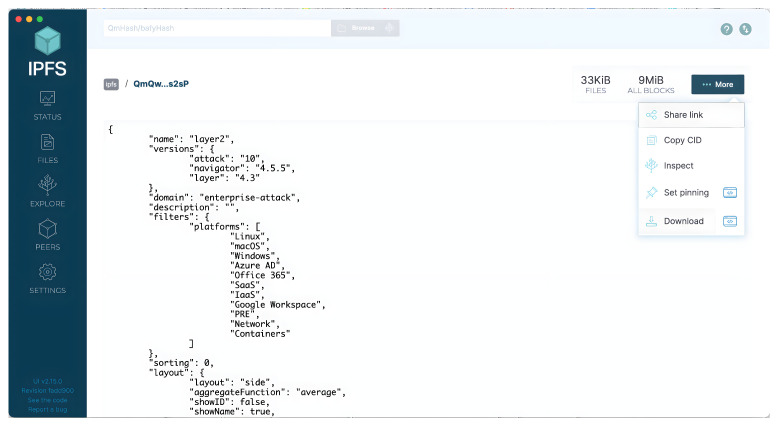
This image depicts the retrieval of the threat information using the obtained CID.

The data are uploaded to the IPFS, as previously stated. As seen in Figure 4, the encrypted file is uploaded and a CID to be shared with the trusted partner is obtained. The CID is then sent to the user who wishes to retrieve the file (User B). User B can access the data map using this CID, as illustrated in Figure 5. Finally, User B can obtain the JSON file and then decrypt it.

#### 4.2.4. Threat Sharing

The proposed infrastructure for threat information sharing has been validated to be highly secure and trusted while providing a large data scale by using Interplanetary File System, encryption techniques, and distributed ledger. Hyperledger Fabric is a distributed ledger, and it represents the proposed ecosystem’s backbone. The steps for developing and running a Hyperledger Fabric network infrastructure are shown in Figure 3.

**Figure 6:**
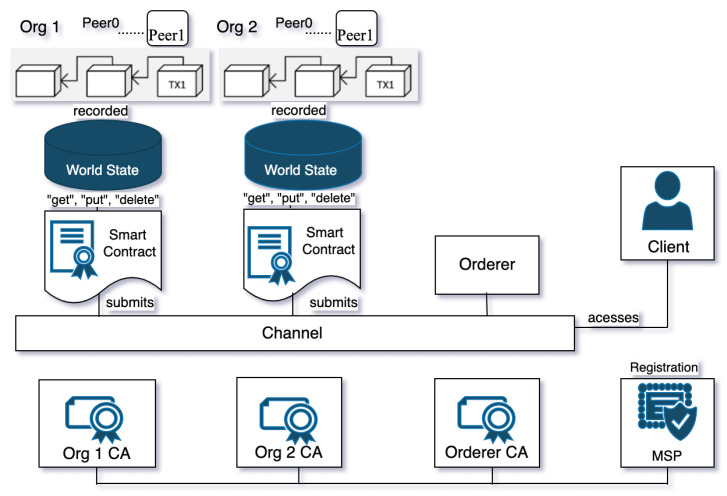
Simple Hyperledger Fabric network that consist of two organisations.

The participants’ nodes are linked through Hyperledger Fabric’s channels. Each authenticated user keeps track of their peers through the chaincode and ledger. This consists of two organisations. Org1 and Org2 are connected through one channel, and each organisation contains two peers, peer 0 and peer 1, as shown in Figure 6. Organisations possibly include different nodes that are connected through one channel or more. Notably, nodes could have multiple chaincodes for storing the transaction data in an immutable ledger.

The client submits the transaction proposal (encrypted JSON file) for endorsement. The transaction is executed by invoking the chaincode functions. Then, the client transactions are endorsed and verified by nodes; only the endorsed transaction is stored in the ledger, and the state of the database is updated. By default, the ledger storage is LevelDB in order to save the ledger’s state, which is available at each peer. In addition, CouchDB is an alternative option that is more efficient for running heavy queries.

The function of each part of the framework and the implementation flow of the system is illustrated in Figure 3 and explained as follows:

1. New threat information is brought from data logs and analysed, classified, and mapped using MITRE ATT&CK. Then, the JSON file is generated. The JSON file is encrypted using self-encryption.
2. User A uploads the encrypted file (chunks) to the IPFS.
3. User A obtains a CID from the IPFS.
4. User A sends the CID to a trustworthy partner. User A (sender) submits the transaction with the CID using their own identity. The sender must be registered and validated using chaincode and endorsement.
5. The trusted partner, User B, must pass the authentication check through the chaincode before obtaining the CID via their identity.
6. User B will use the CID to look for threat files in the IPFS.
7. User B retrieves the desired threat file from the IPFS using the CID.
8. User B decrypts the file to get the JSON file containing threat information.
9. The trusted partner displays the threats events (JSON file) on the MITRE ATT&CK navigator.

We propose our framework that integrates Hyperledger Fabric, IPFS, and MITRE ATT&CK in order to address concerns related to existing and previous threat-sharing frameworks. In response, the proposed ecosystem can securely share threats in a trusted way, since we use encryption techniques and Hyperledger Fabric. Furthermore, the combination of Hyperledger Fabric and the IPFS works to scale up the data storage capacity off-chain. Finally, all of these features make our proposed framework lightweight, and it can accomplish transactions quickly, since we share the hash key (CID) rather than a full-sized threat file embedded within the chaincode of a few asset numbers, as shown in Figure 3.

## 5. System Implementation

The execution of our suggested framework includes the design of use cases, as well as the integration of network infrastructure components. MITRE ATT&CK, the encryption methods, the IPFS storage system, the Hyperledger Fabric network, and smart contracts (chaincode) are the five essential components of the prototype. Consequently, the implemented prototype can be deployed in several phases, as defined in the following subsections.

### 5.1. Threat Intelligence Production Using MITRE ATT&CK

**Figure 7:**
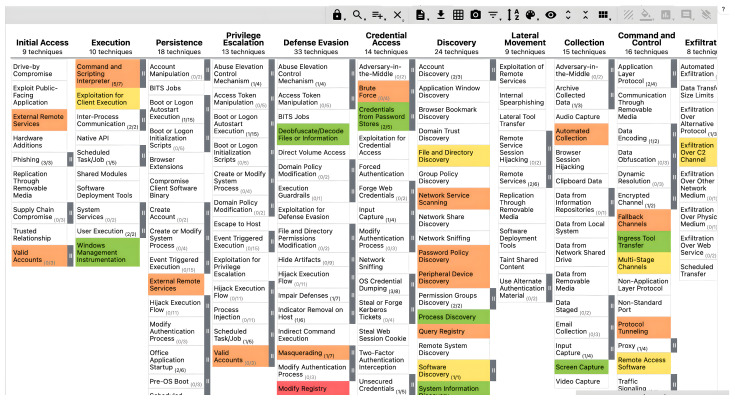
MITRE ATT&CK navigator: The orange colour represents overlapping between multiple threat layers (red, yellow and green).

**Figure 8:**
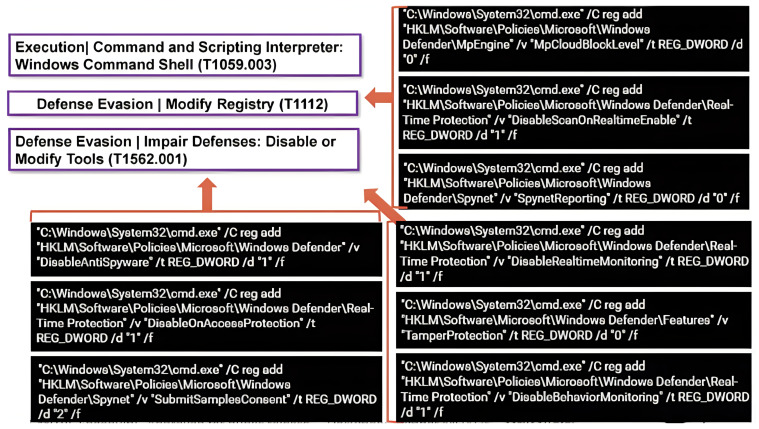
Uncovering new patterns and TTPS through PowerShell.

**Figure 9:**
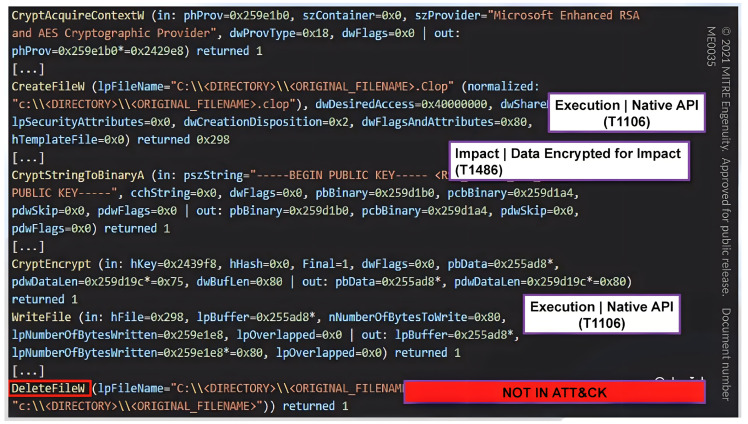
Uncovering new patterns and TTPS through Sysmon.

MITRE ATT&CK, as a vital knowledge base, enables cyber security teams to evaluate and compare attacker activities and then identify the best protection solutions. MITRE ATT&CK is used as a navigator in order to map an attacker’s behavioural data after classifying them to provide valuable information and generate threat intelligence based on past attackers’ tactics and techniques, as shown in Figure 7, Figure 8 and Figure 9, with the following descriptions:

- Tactics: the goals that an attacker is attempting to reach (the column’s title).
- Techniques: the many methods by which cyberattackers can achieve a tactic’s goal (the details under the column).
- Sub-techniques: information that explains the attacker’s technique in further depth.
- Procedures: A process is the particular manner in which a threat actor performs a given technique or sub-technique. A single method can be subdivided into several techniques and sub-techniques.

The overall purpose of employing the MITRE ATT&CK framework is to analyse threats by converting the attacker’s events from data logs into threat information and then into intelligence (tactics, techniques). The MITRE ATT&CK navigator then includes mapping, scoring, and colouring for more straightforward conceptualisation and comprehension. In addition, the MITRE ATT&CK navigator is used to generate a new layer of the threat scenarios, and this layer is downloaded as a JSON file by following the simple aforementioned conceptualisation. Using the MITRE ATT&CK navigator, one can display and review the existing layer, which displays the attacker’s tactics and techniques [34]. These tactics and techniques are included in a JSON file format, which is opened in a layer that shows the threat information contained in the file, as shown in Figure 7. Furthermore, one can integrate two layers of different threats into one layer to analyse them and find the overlapping of varying threat tactics and techniques. For example, the colour orange depicts the overlap between red, yellow, and green. The overlapping layer helps the security analysis team find the most common and repeated tactics and techniques that are being used by attackers.

#### 5.1.1. How Is MITRE ATT&CK Used in Practice?

*Stakeholders:* We need to identify whether threat modelling is acceptable for stakeholders’ use cases. We used tactics and techniques from MITRE ATT&CK to map the events in Figure 8 and Figure 9. A real dataset was used as real evidence, and its resources were collected, classified, and analysed to transfer unstructured data to structured data. Figure 7, shows examples of various threat tactics and techniques that were produced after conducting the data collection and analysis.

#### 5.1.2. System Logs

System logs are the log files in which the system events generated by the components of the operating system are recorded. Their information might range from configuration changes, errors, and updates to device changes, service startup, shutdown, and more. In this context, we will overview a couple of the most prominent monitoring systems at present, PowerShell and Sysmon, as shown in Figure 8 and Figure 9; they are used to convert unstructured data into structured data by transferring them to tactics and techniques by using knowledge based on the MITRE ATT&CK navigator. In order to accomplish automatic and high-quality threat information sharing, several technologies and protocols have emerged for the definition and connection of many different kinds of CTI objects that aim to find a unified language and threat ontology platform. Artificial intelligence (AI) and statistical methods are used to analyse real-time threat event data and convert them into actionable information while considering the unified platforms for trusted partners to share their threat information in an automated and timely manner [28]. MISP is an open-source platform for collecting, storing, analysing, and sharing malware threat information [36]. It used to be called the Malware Information Sharing Platform [37]. MITRE CRITs (Collaborative Research Threats) is a repository for malware and other threats that uses various open-source software tools to form a beneficial and unified program for threat defence analysts and specialists [38]. On top of that, the MITRE Corporation has created the Structured Threat Information Expression (STIX) to assist in the standardisation and sharing of threat information [39]. STIX is discussed as a high-level standard protocol for delivering actionable CTI about threats in the JSON format, making them easy and readable for customers, whether human or machine [40]. Patterns written in the STIX patterning language are more compact and easier to read, since it incorporates additional concepts as they are needed. TAXII is an application-layer protocol for sharing threat information in a simple and scalable manner. TAXII stands for Trusted Automated Exchange of Indicator Information [31]. However, it is not compulsory to use an existing terminology. Therefore, dedicated threat data formats are needed. Integration into current CTI formats will enhance comprehensiveness and efficacy. Thus, threat information sharing and incident response might be fully automated.

In light of the above, we conclude that the threat information cycle includes data collection, analysis, and production in order to transform data into information and then into intelligence to generate meaningful reports and disseminate them. According to the numbering of the tactics and techniques of the new threats reported on the MITRE ATT&CK navigator, one can upgrade the current detection system to be able to deal with the new type of threat. In addition, the system can then export the JSON files that include new threats in order to share them with a trusted partner, as shown in Figure 2 and Figure 3.

The MITRE ATT&CK framework is used to navigate, evaluate, and contrast attacker tactics, techniques, and procedures, as well as to identify the appropriate options for mitigation and countermeasures. Regarding the use-case design, an adversary’s behaviour after being detected is stored in the data log source, then classified to be mapped on the MITRE ATT&CK navigator. These processes aim to transform the data into information and then into intelligence.

### 5.2. Threat Information Sharing through Hyperledger Fabric and Performance Analysis

#### 5.2.1. Environment Setup and Test Configurations

**Figure 10:**
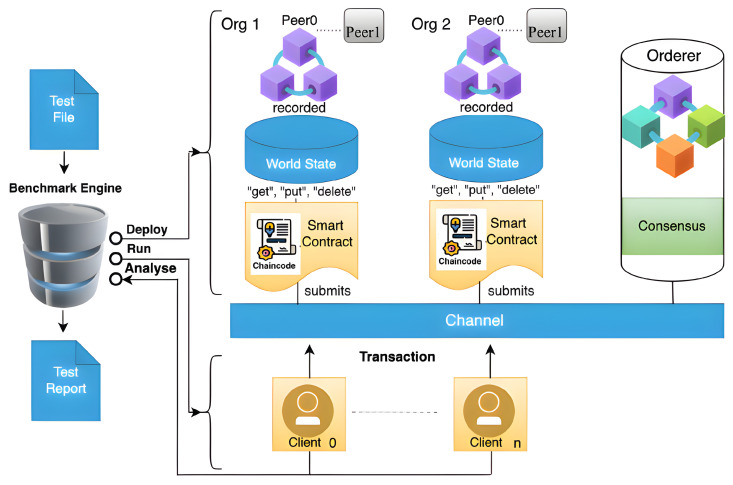
Topology of the benchmark test of Hyperledger Fabric Caliper.

This section includes network performance and functionality tests. First, fabric binaries are installed by using a command line interface to create a two-organisation network, with each having two peers that are connected via a private channel. Then, the benchmark engine interacts with chaincode to deploy, run, analyse, and generate network performance reports, as shown in Figure 10.

The Hyperledger Fabric network setup has prerequisites, as listed in Table 3. (1) The Fabric binary package version 2.2.0 was installed on (2) Ubuntu version 20.04. (3) Docker, version 20.10.7, and (4) Docker Compose, version 1.25.0, were used to generate and operate the entities in the network. Most notably, Hyperledger Fabric, by default, comes with three different optional languages: Golang, Java, and JavaScript. We built the chaincode (smart contracts) using JavaScript (Node.js version 10.19.0).

#### 5.2.2. Interaction System Implementation

The implementation extended the asset-transferbasic/chaincode-javascript formulation by using a *Solo consensus mechanism in the Ordering Service*, which was provided by Hyperledger Fabric and the test network. This performance tested the smart contract on a Fabric network by using Caliper. The basic workflow of this whole system is detailed as follows.

*Implementation of Smart Contracts:* We used Hyperledger Fabric’s smart contracts as a proof of concept (chaincode). The Hyperledger Fabric network was selected because of the features mentioned in the paragraph on the suggested solution. We employed the JavaScript programming language to deploy smart contracts in Hyperledger Fabric’s systems. Finally, we tested it by installing it, approving its definition, committing it to the channel, and invoking the chaincode. By executing the given chaincode, interactions of trusted partners within the Hyperledger Fabric ledger were feasible. Notably, multiple smart contracts could be deployed on the user node.

The chaincode is in charge of dealing with various data queries. As a result, the system implementation began by defining certain chaincode operations, such as querying and retrieving the data lineage. To solve the constraint of threat information sharing in terms of communication speed, storage, and processor power, it was necessary to use the hash key that identified the content address in the IPFS in the implementation of a lightweight chaincode for endorsing peers. The chaincode allowed the trusted partner to get the CID by using their identity to retrieve threat files from the IPFS. In other words, threat information in the IPFS is only accessible to people who have been given permission to use it, since the chaincode can only give out the hash key after a user has been verified. The chaincode is intended to facilitate various data and traceability processes inside the ledger and storage attached to the chain. The proposed system’s chaincode-specific operations include storing data on an item’s world state, querying item checksums, retrieving an object with the relevant transaction ID, extracting the version of an object based on its transaction ID, retrieving the lineage of the data item, retrieving the history of a data object, querying the key range of the list of items (AssetsID), retrieving the threat information, and providing a specific version of an object. Assets represent the variable value of items that may be exchanged on blockchain platforms during transaction execution. The batch size of assets included in the chaincode requested by the others could affect the transaction latency. The key concern is to make the chaincode lightweight so that that the limitation of the file size in threat information sharing can be addressed. Accordingly, we share the hash key and allocate a significant portion of the functionalities to the client applications, such as the Organisation, Owner ID, UserID, CID, Comment, and Colour. The implemented system is made up of distributed peer nodes that serve as the hub for communication among the network parts. The suggested model’s performance was tested in terms of system throughput, send rate, latency, and resource usage (memory, CPU, and network). The scope of the investigation was expanded to examine the latency and scalability of different transaction loads, transaction durations, TPS, and asset batch sizes.

The benchmark involved evaluating ’getAssetsFrom- Batch’ gateway transactions for a fixed-asset smart contract; the endorsement policy was established as a 1-of-any policy, and the network was implemented within the LevelDB and CouchDB state databases. Fabric supports two alternatives for key-value storage; CouchDB and LevelDB were used to maintain the current state. Both are key-value stores; while LevelDB is an embedded database, CouchDB uses a client–server model (accessed using REST API over a secure HTTP) and supports a document/JSON data model. Each transaction obtains a collection of assets from the world state database, which is comprised of a random selection of available UUIDs (universal unique identifiers).

The measurements were carried out with a command-line interface (CLI) by configuring the Caliper benchmarking tool and using the benchmark workspace, network module, and workload to monitor the system’s performance. The test was carried out by simulating a transaction load of 100–2500 through Org1 (“User A”) and Org2 (“User B”), as described in Figure 3. The edge server saved the identities of all connected nodes and authenticated them inside a trustworthy Hyperledger Fabric environment by applying the mutual authentication mechanism described in the Section “II. E. 1”. The suggested model’s performance was evaluated for a variety of workloads and environmental conditions. Furthermore, a diverse set of interaction performances were observed to investigate the improvement or deterioration induced by different model parameters and setups. The benchmarking operations were carried out by using Caliper benchmarking tools that were set to be executed on client nodes, as shown in Figure 10. Because different techniques differ in terms of associated factors and phases, stakeholders frequently need to determine which benchmarking model is appropriate for their applications and specific use cases. We evaluated Hyperledger Fabric V2.2.0 for benchmarking; real-time data reporting and resource consumption statistics were gathered and monitored. The following steps provide examples of various functions through the configuration of the Hyperledger infrastructure and network performance benchmarking.

**Figure 11:**
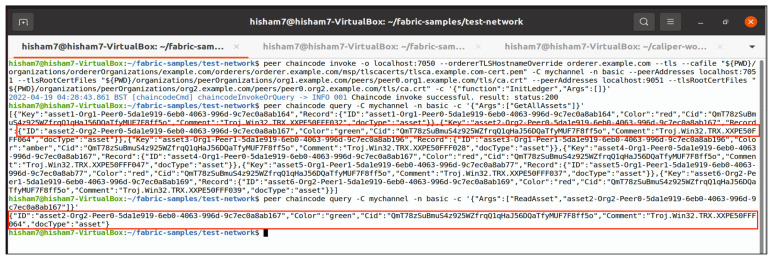
Chaincode invocation with majority endorsement peers.

**Figure 12:**
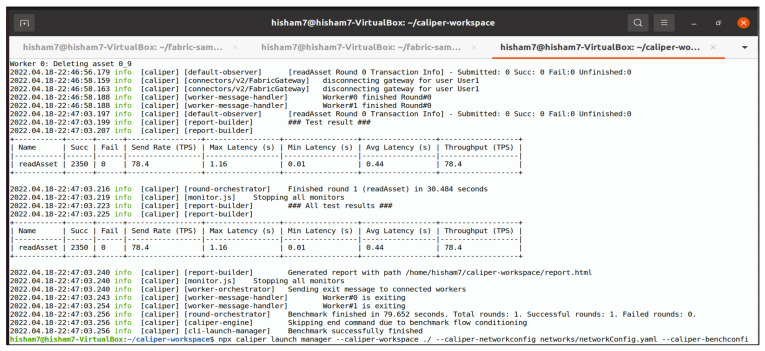
Running the Caliper benchmark and getting the network performance report.

- Bring up the test network and create the channel.
- Package and install the smart contract.
- Approve a chaincode definition.
- Commit the chaincode definition to the channel.
- Invoke the chaincode, as shown in Figure 11.
- Run the Caliper benchmark and get the network performance report by monitoring network latency, send rate, and throughput, as shown in Figure 12.

## 6. Evaluation

Our paper’s focus is on building a reliable ecosystem for disseminating threat information via the use of a permissioned ledger’s data-sharing infrastructure. We tested for aspects such as latency, scalability, security, and anonymity.

**Figure 13:**
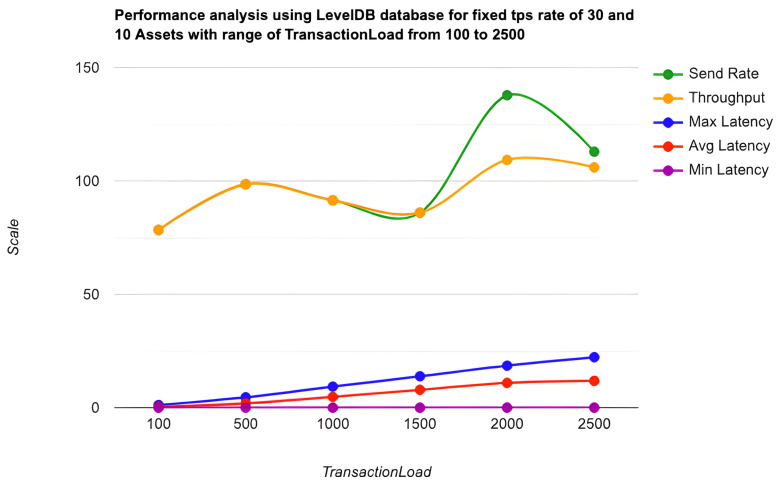
Transaction load using the LevelDB state.

**Figure 14:**
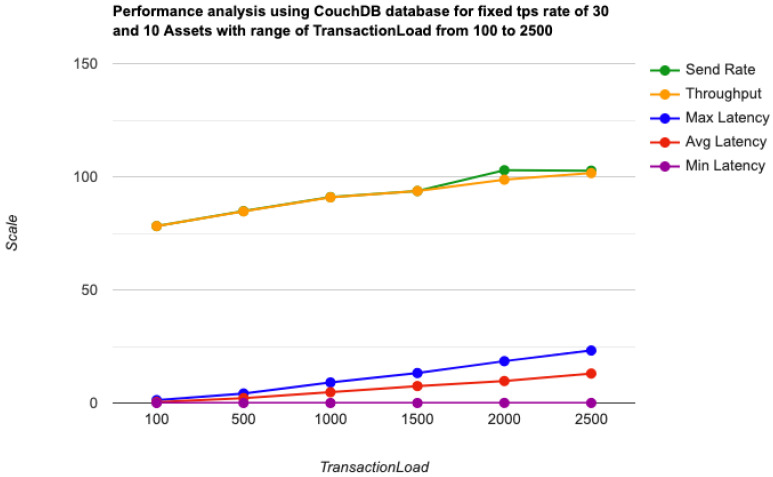
Transaction load using the CouchDB state.

**Figure 15:**
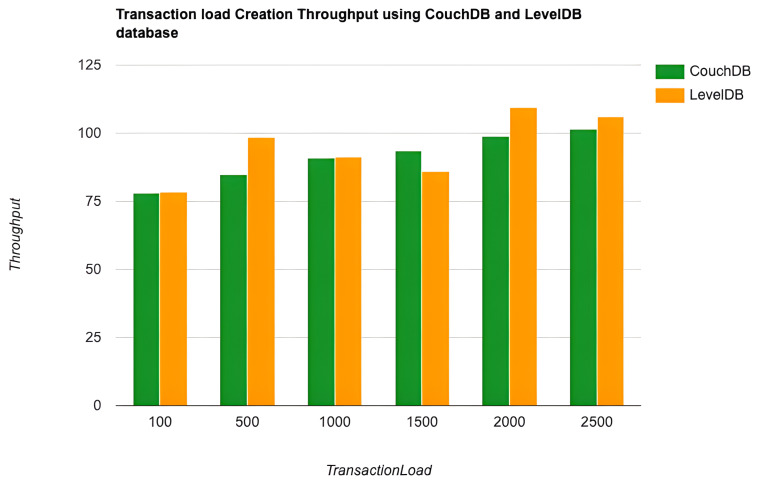
Transaction load creation throughput using the CouchDB and LevelDB databases.

**Figure 16:**
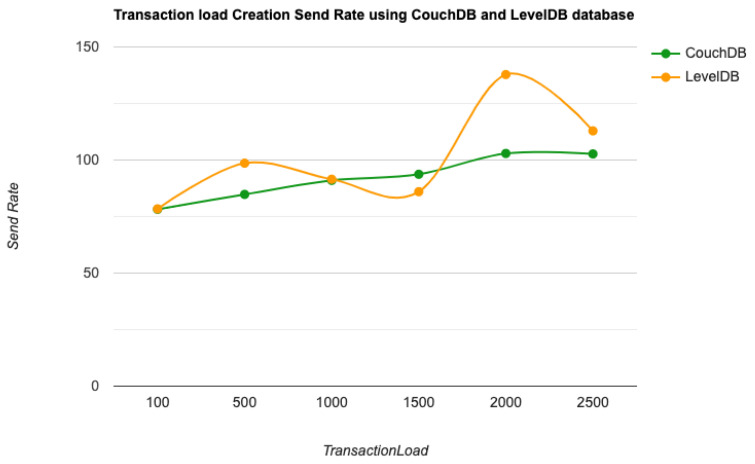
Transaction load creation send rate using the CouchDB and LevelDB databases.

**Figure 17:**
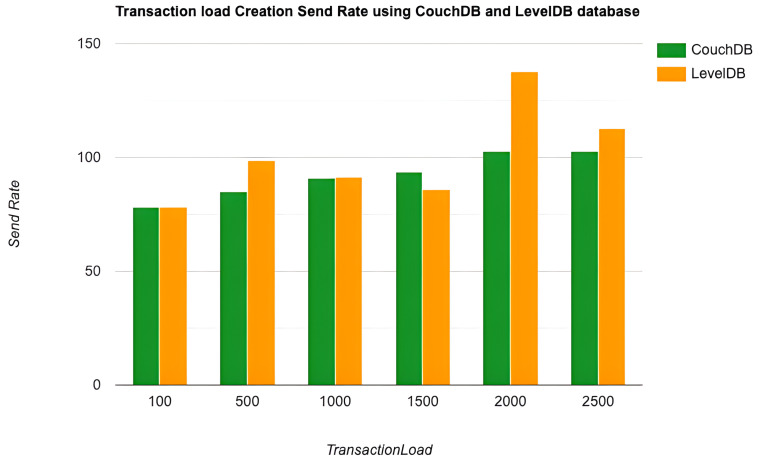
Transaction load creation send rate using the CouchDB and LevelDB databases.

**Figure 18:**
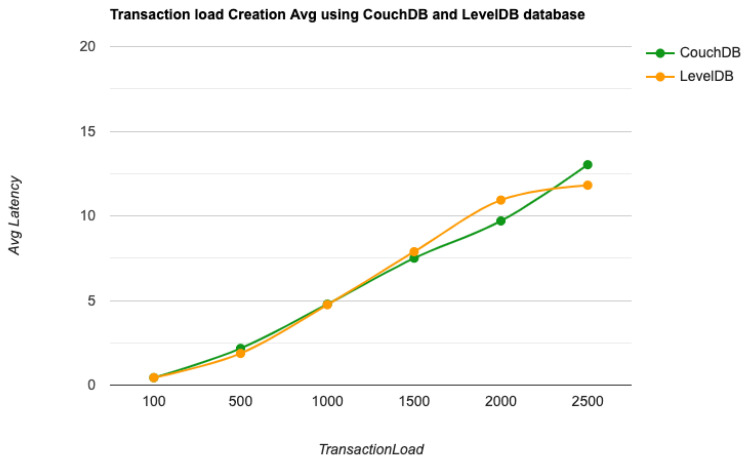
Transaction load creation average latency using the CouchDB and LevelDB databases.

**Figure 19:**
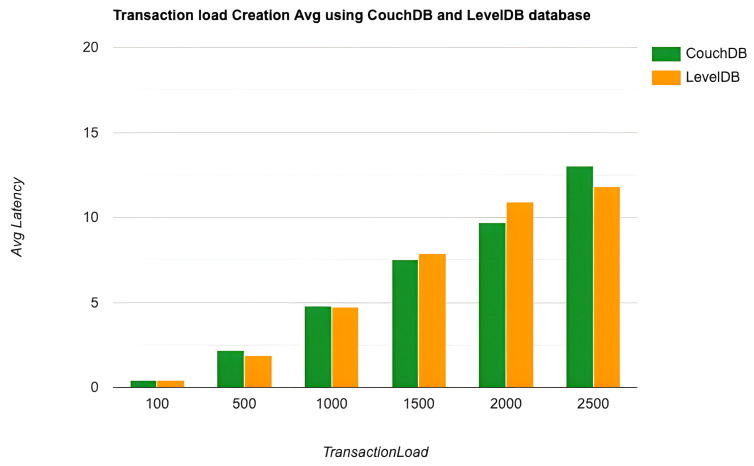
Transaction load creation average latency using CouchDB and LevelDB databases.

**Figure 20:**
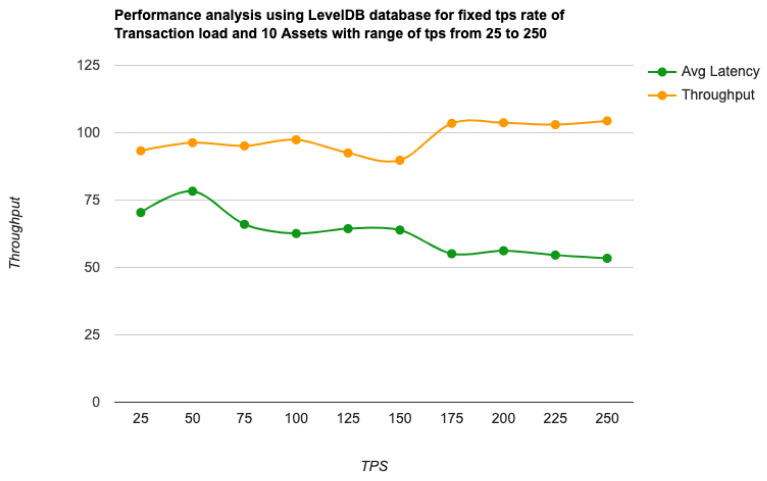
Performance analysis using the LevelDB database for a fixed TPS rate of the transaction load and 10 assets with a range of TPS from 25 to 250.

As the key performance metrics for Fabric, we investigated throughput and latency. The pace at which transactions are committed to the ledger is referred to as the throughput. Latency is defined as the time spent between an application submitting the transaction proposal and the transaction commit, and it is comprised of the following latencies [41]:

- Endorsement latency—the time it took the client to gather all proposal submissions and endorsements.
- Broadcast latency—the period between a client placing an order and the orderer acknowledging it.
- Commit latency—the amount of time it took the peer to verify and commit the transaction.
- Ordering latency—the time spent at the ordering service. This delay is not shown, since the ordering service is not examined in this paper. We also specify three latency blocks:
- Validation system chaincode (VSCC): The validation latency at the VSCC is the time it takes to verify all transactions’ signatures against the policy.
- Multi-version concurrency control (MVCC): The validation latency at this stage is the amount of time required to validate all transactions in a block using multi-version concurrency control.
- Ledger update latency: The latency during the ledger update is the time it takes to update the world state database and create a write-set of all valid transactions in a block.
  Observation 1: Figure 13 and Figure 14 show the analysis of the performance and readings of the Hyperledger Fabric network during transaction executions, as illustrated in Table 4 and Table 5. The send rate was greater than the throughput, and this was a fact. Furthermore, the maximum latency was greater than the average latency, which was a fact too. As shown Figure 15, Figure 16 and Figure 17, when using the LevelDB, throughput (throughput = send rate – packet loss rate) was greater than when using the CouchDB database. This means that the throughput in the LevelDB was better than that in the CouchDB. In contrast, the CouchDB’s latency of reading and writing queries (key value) was better than that of LevelDB.
  Observation 2:
  We can see from Figure 18 and Figure 19 that when we conducted less than 1500 transactions, the latency was better with CouchDB than with LevelDB. However, when we conducted more than 1500 transactions, CouchDB’s latency started to rise sharply and was greater than that of LevelDB.
  Observation 3: We observed from Figure 20 and the resultant Table 6 that, with the increase in the TPS range, the throughput increased linearly, as expected, until it flattened out at a range of 175 to 250 TPS. The saturation point was 175. The latency increased slightly when the arrival rate was close to or above the saturation point, where it flattened. One can conclude that the number of ordered transactions in the validation system chaincode (VSCC) queue increased quickly during validation, and the commit latency increased. This was due to the fact that the execution phase was independent from the validation phase. In other words, since the VSCC only employed a single virtual central processor unit (vCPU), new transaction proposals relied on additional vCPUs at the peer for simulation and validation. As a consequence, the validation process was the only one that became a bottleneck. We can summarise several positive, valuable points from our experiments through our three observations, which could help us find answers to the questions being raised, such as that of the transaction load that would lead to the bottleneck issue at the validation phase. Undoubtedly, the answer will help software developers work on this issue and understand the best parameters, databases, processors, memory, and consensus algorithm types to determine the best practices and provide better performance for our network regarding the send rate, latency, and throughput.

### 6.1. Security and Availability Considerations

Our proposed design is a distributed network that is resistant to DoS attacks, since there is no central server. Instead, data are equally distributed among all nodes. Furthermore, Hyperledger Fabric offers transaction encryption and chaincode, which control the interactions between trusted users while providing transaction authentication and data secrecy. Every user must pass an identity verification process; otherwise, their transaction will be denied. Furthermore, as part of Hyperledger Fabric, which provides certificate authority and identity management, we employ the MSP entity, which is one of the core components that provides system authentication. This entity offers unique identities for all nodes and channels, allowing transactions between interacting users to remain private and confidential while traversing the network. The system is secured against unauthorised users by default. The human factor might be a possible risk in our suggested system, since users’ credentials could be intentionally or unintentionally hacked.

Accordingly, the certificate authority is the third party that establishes a trusting relationship between the interacting parties by its signature being used as evidence of identity. If one of the parties in a transaction is compromised, that party might ask the certificate authority to revoke all of their previous transactions. As a result, an attacker attempting to access Hyperledger Fabric via reading or writing queries will be exposed, since it needs system IDs. The only way is to breach the identity of an authorised user. Another possible issue is that adversaries may target blockchain ledgers, since they are distributed across participating nodes, preventing normal users from accessing them.

### 6.2. Anonymity Considerations

Because it contains the MSP entity, which is in charge of identity management, Hyperledger Fabric provides transaction anonymity by default. Furthermore, this component is pluggable, which means that each organisation can establish its own MSP. Transaction data anonymity is derived from transaction data encryption, which is performed with the client’s private key and signed by a trusted third party (identity management) and the transport-layer security cryptographic protocol (TLS), which is used to encrypt communication between interacting participants. An authorised user will be recognised in the chaincode and will need to collect enough endorsements to be qualified to send the transaction for validation, which is regulated by the endorsers’ rules, without releasing or revealing the transaction details.

The authors of [42] suggested an *anonymous endorsement system* with a threshold endorsement policy in their work. Several considerations led to the development of a novel ring signature scheme known as Fabric’ Constant-Sized Linkable Ring Signature (FCsLRS) with transaction-oriented linkability for concealing the identities of the endorsers. Furthermore, by altering the RSA (Rivest–Shamir–Adleman) modulus size, this study built the signature technique in Golang and examined its security and performance. An empirical study backs up the viability of the implementation. The production of signatures and tags is relatively quick. Furthermore, a given RSA modulus value remains constant regardless of the message length or endorsement set size, provided that all endorsers create their signatures in tandem.

### 6.3. Latency, Scalability, and Throughput

The latency of reading and writing queries in Hyperledger Fabric is similar to that in traditional centralised systems, such as standard databases (PostgreSQL), rather than in public blockchain systems, such as Bitcoin. It is important to note, however, that our chosen architecture outperforms standard local databases in terms of efficiency as transaction volumes increase [3].

Hyperledger Fabric leverages Docker containers as blockchain nodes, making it readily scalable to cloud infrastructures, such as Kubernetes clusters. This significantly increases the stated use cases by combining several peers and organisations to build complicated situations. Furthermore, the actual records can be replicated through IPFS nodes in our suggested ecosystem, extending the situation even further.

The ordering service determines our system’s transaction throughput and is limited by the network and ordering node capacity. However, the transaction throughput may be enhanced by simply increasing the number of ordering nodes. RAFT and KAFKA ordering clusters are two examples of the addition of extra nodes [22].

## 7. Conclusions and Future Work

Threat hunting is primarily a proactive countermeasure to identify and safeguard IT systems from hostile behaviour by monitoring emerging and existing threats. As a result, a reliable and secure infrastructure for sharing threat information is required. The use of data logs created by systems, network devices, or security applications, such as intrusion detection/prevention systems, can assist in achieving this aim by deploying trusted computing technologies for adversary detection and privacy protection. Our study presented a trusted threat information sharing solution that uses the IPFS system and a permissioned ledger to maintain the security, privacy, and anonymity of stored data while achieving a fast throughput and huge scalability. Hyperledger Fabric is the permitted distributed ledger technology of our choice, since it satisfies all of the standards mentioned above and achieves the goals of our work. When a set of defined conditions are met, our proposed infrastructure effectively implements threat information sharing by utilising the MITRE ATT&CK framework, pluggable certificate authorities, and self-executing chaincode, enabling trust between the interacting trusted parties and enhancing the overall security of the system. As a result, our ecosystem assures that the stored information remains anonymous. The Hyperledger Fabric technology has security flaws. These limits might be overcome. Future research may incorporate malicious nodes that would affect our model. Solo may not be secure enough for production usage. In the near future, we plan to develop a comprehensive proof-of-concept in a cloud architecture utilising a Kubernetes cluster to increase the system’s throughput and scalability. We will also increase the system workload by expanding the number of participating nodes and the number of transactions.

## Figures and Tables

**Table 1:** Comparison of different blockchain types.

Comparison Features	Public Blockchain (Permissionless)	Private Blockchain	Permissioned Blockchain
**Read**	It is an open network, no permission needed, anyone can download protocol and read	Only specific participants in the organisation can read, verify, and add new nodes	Under a legal contract, the public and participants are permissible
**Write**	It is an open network, no permission needed, anyone can download protocol and write	Only specific participants in the organisation can write	Participants are permissible under some legal contracts
**Consensus Operational**	No conditions are needed to join consensus; the process needs more energy and resources.	Only those who are pre-selected can conduct the consensus within the organisation	Pre-selected nodes within consortium
**Examples**	***Bitcoin***	***Ethereum***	***Hyperledger Fabric***
**Network**	Permissionless	Permissioned or Permissionless	Permissioned
**Classification**	Public	Public or Private	Private
**Governance**	Decentralised	Ethereum Developers	Linux Foundation
**Currency**	Yes	Yes - Ether To-kens (smart contract)	Non-Currency Tokens (chaincode)
**Operation Pattern**	Order–execute	Order–execute	Execute–order–execute
**Cost**	Yes, Satoshi (It is synonymous with Bitcoin).	Yes, Gas (the amount of computational power).	None
**Smart Contracts**	No	Smart Contracts (Solidity, Serpent, LLL)	Chaincodes (Go, JavaScript, Java, and more)
**Consensus Algorithms**	Proof of Work	Proof of Work or Proof of Stake (new versions)	Normal operation or Practical Byzantine Fault Tolerance (PBFT)
**Encryption of transaction data**	No	No	Yes
**TPS**	3.3–4.6	15	Up to 5000
**Block Size**	1–2 MBs 4 MB SegWit (Segregated Witness)	20–30 KB	By default: 512 KB (Preferred), 98 MB (Absolute Maximum)
**Transactions per Block**	3500	70	10 (default)
**Block Time**	10 min	15 s	1 s
**Currency Capitalisation**	21 million	5 every 14 s	No Currency
**Current Block Reward**	12.5 BTC	3 ETH	N/A
**Applications**	Track ownership of Digital Currency (Mostly)	DApps (Games, IoT, Fintech, Supply Chain, and so on)	Private Blockchain requires high performance, resiliency, and privacy

**Table 2:** Comparison of the related literature with our work.

Related Work	Type of Blockchain	Governance	Cost	Scalability	Privacy	Integrity	Anonymity
Bawane et al. [24]	Ethereum	No	Yes	Yes, IPFS	No	Yes	No
Havelange et al. [25]	Public Blockchain	No	No	No	No	Yes	No
Wang et al. [26]	Public Blockchain	No	Yes	Yes, Blockchain	Yes	Yes	Proxy Encryption
Politou et al. [27]	Private Blockchain	No	Yes	Yes, IPFS	Yes	Yes	Encryption
Preuveneers et al. [28]	Distributed Ledger	Yes	Yes	Yes	Yes	No	CP-ABE
Grundstrom et al. [29]	Hyperledger Fabric	Yes	None	Yes, IPFS	Yes	Yes	User Identity
Our Work	Hyperledger Fabric	Yes	None	Yes, IPFS	Yes	Yes	User Identity + Self-Encrypted

**Table 3:** Setup of the Hyperledger Fabric Network.

System & Tools	Version
Operating System	Ubuntu 20.04
Hyperledger Fabric	2.2.0
Docker	20.10.7
Docker-compose	1.25.0
Node.js	10.19.0

**Table 4:** Transaction load using the LevelDB state.

Transaction Load	Send Rate (TPS)	Max Latency (s)	Min Latency (s)	Avg Latency (s)	Throughput (TPS)
100	78.4	1.16	0.01	0.44	78.4
500	98.6	4.59	0.02	1.87	98.5
1000	91.5	9.30	0.01	4.75	91.4
1500	86.6	13.87	0.01	7.88	86.0
2000	137.8	18.52	0.03	10.93	109.3
2500	112.9	22.28	0.03	11.81	106.0

**Table 5:** Transaction load using the CouchDB state.

Transaction Load	Send Rate (TPS)	Max Latency (s)	Min Latency (s)	Avg Latency (s)	Throughput (TPS)
100	78.2	1.29	0.01	0.44	78.2
500	84.8	4.22	0.01	2.17	84.7
1000	91.0	9.07	0.01	4.78	90.9
1500	93.7	13.23	0.01	7.50	93.7
2000	102.9	18.50	0.05	9.70	98.7
2500	102.7	23.20	0.03	13.02	101.6

**Table 6:** Performance analysis using the LevelDB database for a fixed TPS rate of the transaction load and 10 assets with a range (txDuration) from 25 to 250.

Transaction Duration	Send Rate	Max Latency	Min Latency	Avg Latency	Throughput
25	106.3	13.96	0.02	7.04	93.3
50	96.4	14.96	0.01	7.83	96.3
75	95.1	13.26	0.03	6.60	95.1
100	97.4	13.16	0.02	6.26	97.4
125	92.5	13.63	0.02	6.44	92.5
150	89.8	13.39	0.01	6.39	89.8
175	103.5	12.80	0.02	5.51	103.5
200	103.7	14.66	0.03	5.62	103.7
225	103.0	14.78	0.01	5.46	103.0
250	104.4	12.22	0.02	5.34	104.4

## Data Availability

Data sharing is not applicable for this article.
